# Dose Optimisation of Posaconazole and Therapeutic Drug Monitoring in Pediatric Patients

**DOI:** 10.3389/fphar.2022.833303

**Published:** 2022-04-19

**Authors:** Mengmeng Jia, Qiwen Zhang, Zifei Qin, Dao Wang, Peng Liu, Jing Yang, Xiaojian Zhang

**Affiliations:** ^1^ Department of Pharmacy, The First Affiliated Hospital of Zhengzhou University, Zhengzhou, China; ^2^ Henan Key Laboratory of Precision Clinical Pharmacy, Zhengzhou University, Zhengzhou, China; ^3^ Department of Pediatrics, The First Affiliated Hospital of Zhengzhou University, Zhengzhou, China; ^4^ Pediatric Intensive Care Unit, The First Affiliated Hospital of Zhengzhou University, Zhengzhou, China

**Keywords:** posaconazole, pediatric patients, invasive fungal infections, therapeutic drug monitoring, pharmacokinetics

## Abstract

Experience in the clinical use of posaconazole (PCZ) in pediatric patients is limited, and no specific dose recommendations exist. This study aimed to investigate an appropriate dosing regimen, and assess the exposure-response relationships of PCZ in children. We reviewed the medical records of inpatients aged <18 years who subjected to PCZ concentrations monitoring. Clinical data, PCZ dosing and monitoring data were collected. A total of 375 PCZ trough concentrations (*C*_min_) from 105 pediatric patients were included. For children receiving PCZ for prophylaxis, the median doses required to achieve the therapeutic range at the ages of <6, 6–12 and >12 years were 14.80, 14.52 and 12.90 mg/kg/day, respectively (*p* = 0.001); and for those receiving PCZ for treatment, the median doses were 23.50, 20.96 and 15.38 mg/kg/day, respectively (*p* = 0.001). Among children taking PCZ for prophylaxis, 12% developed a proven or probable breakthrough IFIs; the median PCZ concentrations were significantly lower than those children with successful treatment response (0.43 versus 1.20 μg mL^−1^; *p* < 0.001). 79.2% patients taking PCZ for treatment had a positive clinical response, and the median PCZ concentrations were significantly higher than those children with disease progression (1.06 versus 0.53 μg mL^−1^; *p* = 0.024). No association between *C*_min_ values and hepatotoxicity was observed. Factors such as age, CRP, ALT and co-administration with proton pump inhibitors exhibited significant effects on PCZ *C*_min_. It is necessary to adjust the dosing regimens based on PCZ *C*_min_ to individualize antifungal therapy and provide guidelines for dose adjustment in children.

## Introduction

Invasive fungal infections (IFIs) represent a major cause of morbidity and mortality in immunocompromised children (Lehrnbecher and Groll, 2011). Posaconazole (PCZ) is an extended-spectrum triazole antifungal used as prophylaxis of and treatment for IFIs in these patients (Groll et al., 2014; Arrieta et al., 2019). Several studies have demonstrated a correlation between PCZ plasma levels and clinical efficacy (Jang et al., 2010; Moore et al., 2015). Therefore, therapeutic drug monitoring (TDM) of PCZ can improve its efficacy and safety (Ashbee et al., 2014; Chau et al., 2014; Döring et al., 2017). Our previous research which conducted in adults demonstrated that a number of factors exhibited significant effects on PCZ *C*_min_ including gender, albumin and interactions with co-medications, leading to high inter-individual and intra-individual variability in plasma concentrations. A 25.9% lower in the exposure of the oral PCZ suspension was reported when administered with proton pump inhibitors (PPIs) in adults (Dolton et al., 2014; Jia et al., 2020). However, fewer data are available in children and most of them are based on adult experience for pediatric patients.

Individual differences in concentrations of this drug in children blood serum are greater than adults, achieving and maintaining therapeutic concentrations for the duration of therapy is difficult due to the drug interactions and erratic absorption in PCZ pharmacokinetics. thus, the monitoring of trough concentrations in children is of the utmost importance for PCZ therapeutic effectiveness and safety (Jancel et al., 2017; Boonsathorn et al., 2019). There is an agreement that children need higher and personalized doses to reach therapeutic *C*_min_ (McMahon et al., 2017; Lai et al., 2020). It is difficult to attain the target concentration of PCZ for children and there is a significant variability in the dose required because of its unpredictable bioavailability (Gwee et al., 2015). To date, there is few published recommendations on PCZ dosing for pediatric patients.

Therefore, we performed a retrospective analysis to investigate a potential relationship between PCZ *C*_min_ and its efficacy and safety among children, and explore an appropriate dosing regimen as well as the potential factors influencing PCZ *C*_min_ in children.

## Methods

### Patients’ Enrollment and Data Collection

This retrospective study was performed in The First Affiliated Hospital of Zhengzhou University (Zhengzhou, China). Pediatric patients (age <18) taking PCZ oral suspension for treatment or prophylaxis of IFIs and performing at least one PCZ concentration determination during therapy between June, 2018 and December, 2020 were enrolled in this study. Medical records and demographic information of these children were reviewed. Details of PCZ therapy, outcomes of therapy, adverse events, concomitant medications that could potentially interfere with PCZ absorption, and biochemical parameters including CRP, albumin, alkaline phosphatase (ALP), aspartate aminotransferase (AST), alanine aminotransferase (ALT), g-GT (gamma-glutamyl transferase), and bilirubin values were also collected.

### PCZ Regimen and Therapeutic Drug Monitoring

The blood samples for measuring PCZ *C*_min_ were collected for routine care. Only children having achieved steady-state were included in the analysis and PCZ was considered to have achieved a steady-state plasma concentration after at least 7 days of dosing (Lai et al., 2020). Dose adjustments were taken according to the current guidelines and the manufacturer’s recommendations (Vicenzi et al., 2018). PCZ trough plasma concentrations were determined using a previously described ultra-high-performance liquid chromatograph-tandem mass spectrometry method (UPLC-MS/MS, Waters, United States) (Jia et al., 2020). The analytical range was 0.025–5.00 μg mL^−1^. For the purpose of this study, plasma concentrations ≥0.7 μg mL^−1^ were considered therapeutic for prophylaxis and ≥1.0 μg mL^−1^ for treatment (Bernardo et al., 2020).

### Diagnostic Criteria

Only patients with a PCZ treatment regimen of ≥14 days were incorporated into the study to assess clinical response. Fungal infections and the response to antifungal therapy were retrospectively classified according to the definitions of the European Organization for Research and Treatment of Cancer/Mycoses Study Group (EORTC-MSG) (Pauw et al., 2008). For patients receiving PCZ for prophylaxis, it would be assessed as a positive outcome if the course was completed without breakthrough fungal infection (Pauw et al., 2008; Chinese Association Hematologists; Chinese Invasive Fungal Infection Working Group, 2020). For patients receiving PCZ for treatment, of a fungal infection, a successful outcome was defined as partial improvement or improvement of clinically significant signs and symptoms, improvement or resolution of radiological signs of infection and evidence of microbiological cure; otherwise, patients were classified as clinical failure. Adverse drug reactions and their relationship with PCZ were defined according to the Common Terminology Criteria for Adverse Events (CTCAE) defined by the National Cancer Institute (National Cancer Institute, 2006).

### Statistical Analysis

Statistical analysis was performed with SPSS Statistics for Mac Ver. 26 (SPSS Inc., Chicago, IL). Patients were divided into three groups (<6, 6–12, and >12 years old) according to previous literature (Döring et al., 2017; Lavigne et al., 2019). Descriptive statistics included the mean, standard deviation, and median. The categorical variables were expressed as frequency and percentage. Categorical variables were compared using *χ* ^2^ test or Fisher’s exact test. Continuous variables were compared using Mann-Whitney U-test or Kruskal-Wallis H test. The correlation between two continuous variables was examined using the Spearman correlation coefficient. The association of *C*_min_ with efficacy and safety was analyzed by logistic regression. Affecting factors of PCZ trough concentrations were analyzed using multivariate analysis by linear regression. *p* values < 0.05 were considered statistically significant.

## Results

### Patient’ Characteristics

**FIGURE 1:**
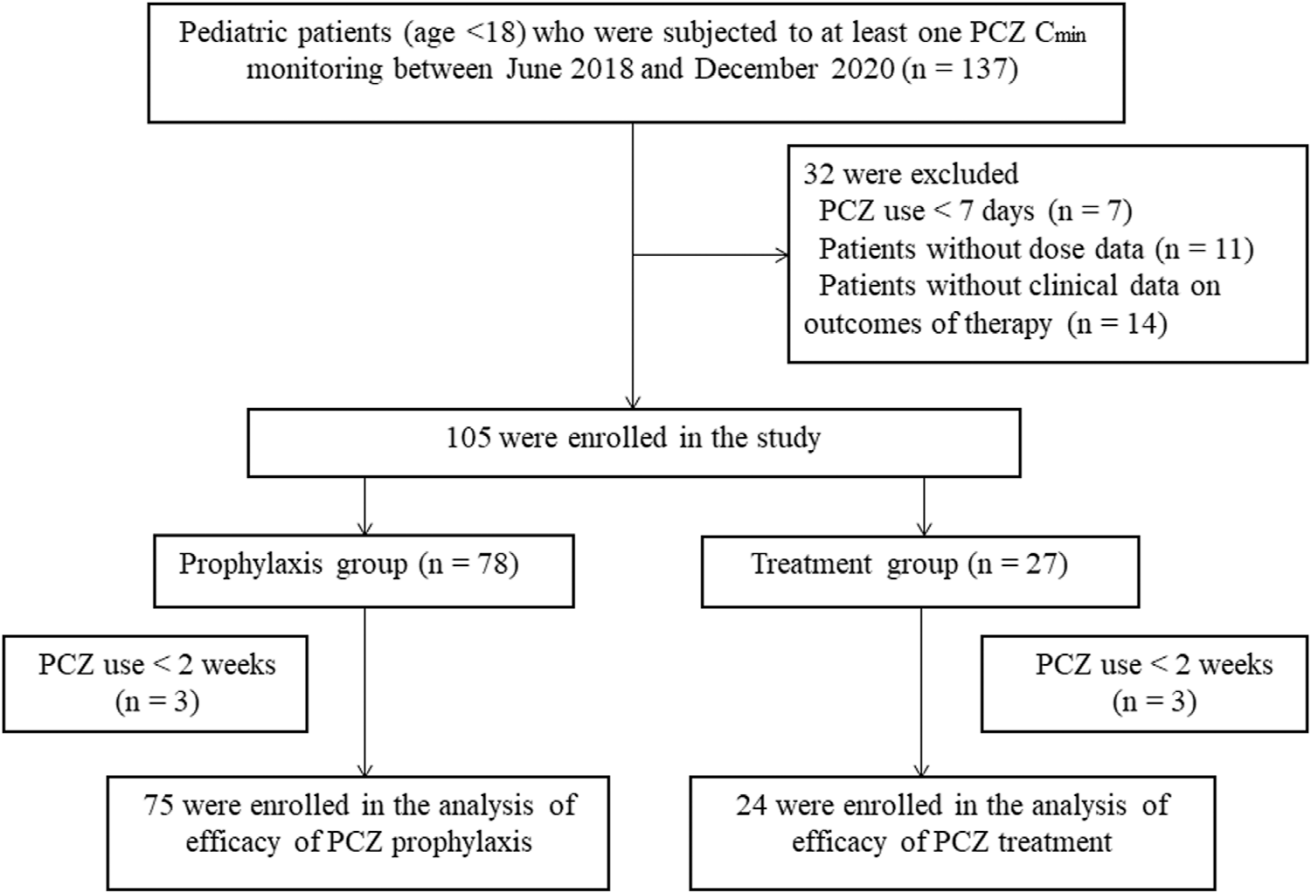
Flow chart of the study.

A total of 375 PCZ *C*_min_ drawn in 105 pediatric patients were available in this research (Figure 1) 32 (30.5%) females and 73 (69.5%) male patients were included, with a median age of 10 years (range, 1.5–18 years). The most frequent underlying condition was acute lymphoblastic leukaemia (34.29%). Seventy-two (68.6%) received an allogeneic hematopoietic stem cell transplantation (HSCT) before using PCZ. A median of 2 trough levels (range, 1–15) were measured per patient, and the median therapy duration of all patients was 84 days (range, 7–320 days). Demographic information of these patients is summarized in Table 1.

**TABLE 1:** Patient characteristics.

Characteristics	Total (*n* = 105)	<6 years	6–12 years	>12 years
		*n* = 31 (29.5%)	*n* = 32 (30.5%)	*n* = 42 (40.0%)
Age (years)	10 (1.5–18)	3 (1.5–5)	9 (6.0–12)	16 (13.0–18)
Weight (kg)	32.9 (7.5–95)	14.8 (7.5–20.2)	28.4 (16.0–44.0)	51.5 (36.5–95.0)
BMI (kg/m^2^)	16.0 (11.45–28.73)	14.86 (11.45–22.50)	15.10 (12.10–20.41)	18.0 (14.55–28.73)
Male	73 (69.5)	22 (71.0)	17 (53.1)	34 (81.0)
HSCT	73 (69.5)	17 (54.8)	26 (81.3)	30 (71.4)
Days of use (days)	84 (7–320)	66 (8–238)	116.5 (8–310)	80 (7–320)
Numbers of measurements	2 (1–15)	3 (1–11)	2 (1–15)	2.5 (1–14)
PCZ concentration				
Cmin (ug·mL^−1^)	1.07 (0.04–4.84)	1.05 (0.11–2.71)	1.08 (0.06–4.07)	1.08 (0.04–4.84)
Cmin/dose (ug·mL^−1^·g^−1^)	2.33 (0.05–18.25)	3.87 (0.36–18.25)	2.70 (0.11–10.18)	1.36 (0.05–6.25)
Treatment Indication				
Therapeutic	27 (25.7)	8 (25.8)	5 (15.6)	14 (33.3)
Prophylactic	78 (74.3)	23 (74.2)	27 (84.4)	28 (66.7)
Underlying conditions				
AML	31 (29.5)	10 (32.3)	10 (31.3)	11 (26.2)
ALL	36 (34.3)	9 (29.0)	8 (25.0)	19 (45.2)
Lymphoma	5 (4.8)	3 (9.7)	1 (3.1)	1 (2.4)
AA	23 (21.9)	5 (16.1)	8 (25.0)	10 (23.8)
MDS	2 (1.9)	0 (0.0)	2 (6.3)	0 (0.0)
HPS	2 (1.9)	0 (0.0)	2 (6.3)	0 (0.0)
Other	6 (5.7)	4 (12.9)	1 (3.1)	1 (2.4)

Data is presented as n (%), or as median (range). BMI, Body mass index; Cmin, rough plasma concentration; AML, acute myeloid leukaemia; ALL, acute myeloid leukaemia; AA, Aplastic anemia; MDS, myelodysplastic syndrome; HPS, hemophagocytic syndrome.

### PCZ Plasma Concentrations

**FIGURE 2:**
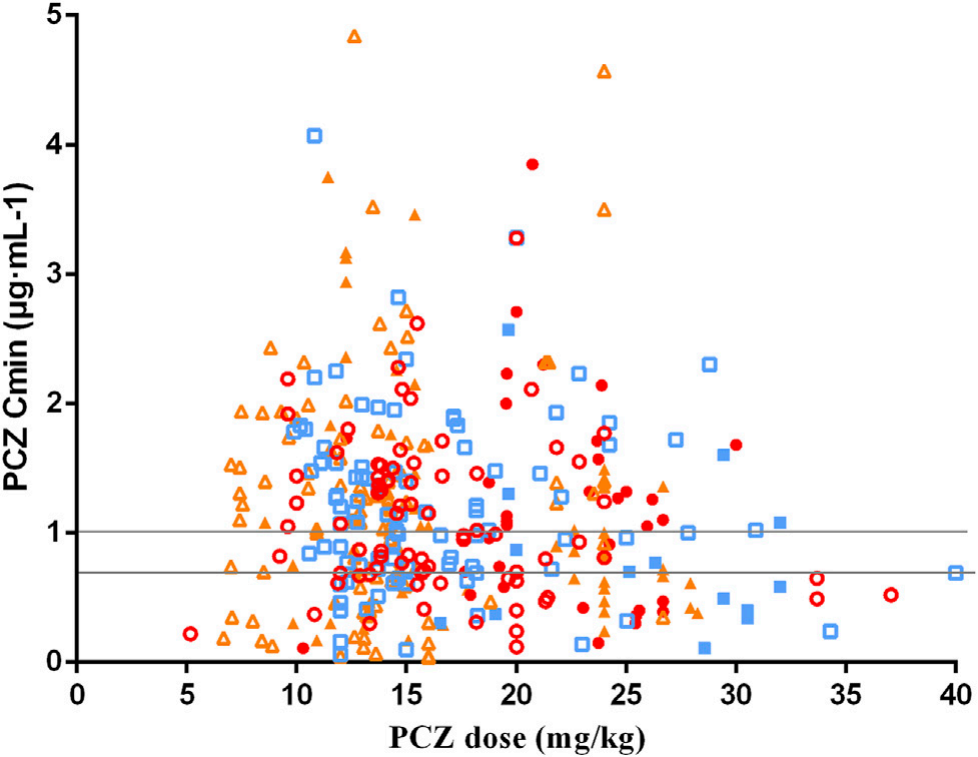
Distribution for PCZ Cmin at different weight-adjusted doses received. Solid and hollow red circles represent the data obtained of PCZ prophylaxis and treatment in children aged <6 years old, respectively. Solid and hollow blue square represent the data obtained of prophylaxis and treatment in children aged 6–12 years old, respectively. Solid and hollow orange triangles represent the data obtained of prophylaxis and treatment in children aged >12 years old, respectively. Two horizontal lines indicates the therapeutic range of PCZ prophylaxis and treatment (0.7 μg·mL-1 and 1.0 μg·mL-1).

The median PCZ plasma concentration for all 375 trough levels was 1.07 μg mL^−1^ (range, 0.04–4.84 μg mL^−1^; mean 1.16 ± 0.76 μg mL^−1^). Timing of the first TDM sample varied widely, with a median of 7 days (range, 7–34 days) after starting therapy. Distribution of PCZ trough concentrations is shown in Figure 2.

Seventy-eight patients (74.3%) received PCZ for prophylaxis with 273 PCZ plasma concentration measured. The median PCZ plasma concentration was 1.10 μg mL^−1^ (range, 0.04–4.84 μg mL^−1^; mean 1.18 ± 0.73 μg mL^−1^). The median duration of therapy was 51 days (range, 8–306 days). 74.4% of these patients had therapeutic serum concentrations (≥0.7 μg mL^−1^). There is no significant difference in the number of PCZ *C*_min_ reaching the target concentration among the three age groups (<6 ages, 71.43%; 6–12 ages, 79.79%; >12 ages, 71.57%; *χ* ^2^ = 2.216, *p* = 0.333). Twenty-seven (25.7%) received PCZ for treatment with 102 PCZ plasma concentration measured. The median PCZ *C*_min_ was 0.91 μg mL^−1^ (range, 0.07–3.85 μg mL^−1^; mean 1.11 ± 0.84 μg mL^−1^). 48.04% of these patients had therapeutic concentrations (≥1.0 μg mL^−1^). No significant difference in the number of PCZ *C*_min_ within the therapeutic range among the three age groups were observed (<6 ages, 56.25%; 6–12 ages, 35.29%; >12 ages, 47.17%; *χ* ^2^ = 1.987, *p* = 0.370).

**FIGURE 3:**
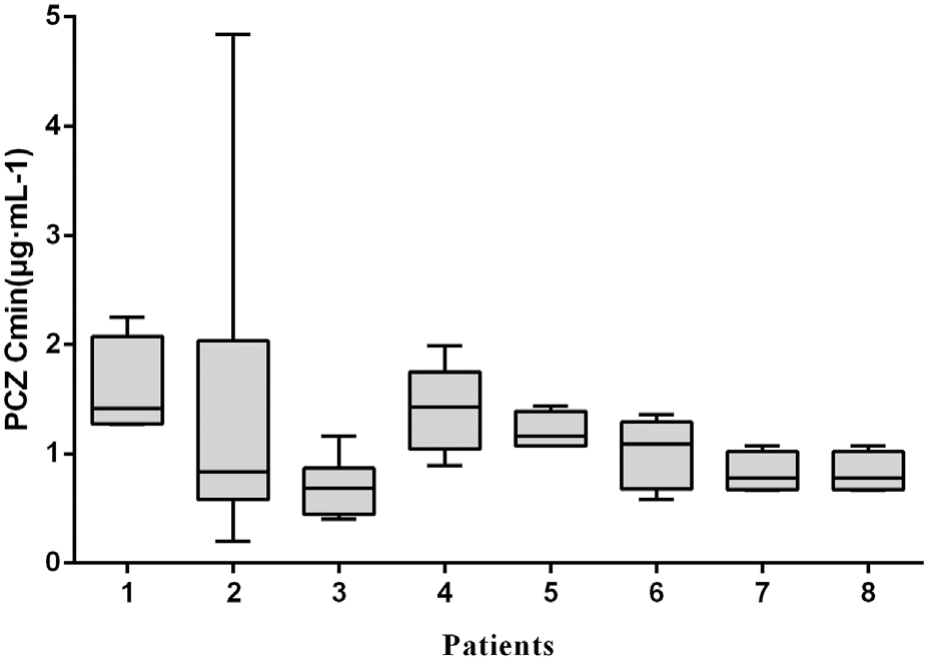
Intra-and inter-patient variability of PCZ Cmin. Each boxplot represents PCZ levels (median, minimum, maximum and interquartile range) of a single child for a total of 8 children receiving 12 mg/kg/day of PCZ suspension who had five or more sample measurements.

Moreover, there was no significant correlation between PCZ *C*_min_ and weight-adjusted dosage for all children <6, 6–12 and >12 years of age, regardless of the indication of treatment (*p* > 0.05 for all 6 groups). Large intra- and inter- patient variability for patients receiving 12 mg/kg/day of PCZ suspension who had five or more sample measurements was observed (Figure 3), and the median intra-patient variability of these children was 28.75% (range, 12.89–119.83%).

### Maintenance Dose to Achieve the Target Range

**FIGURE 4:**
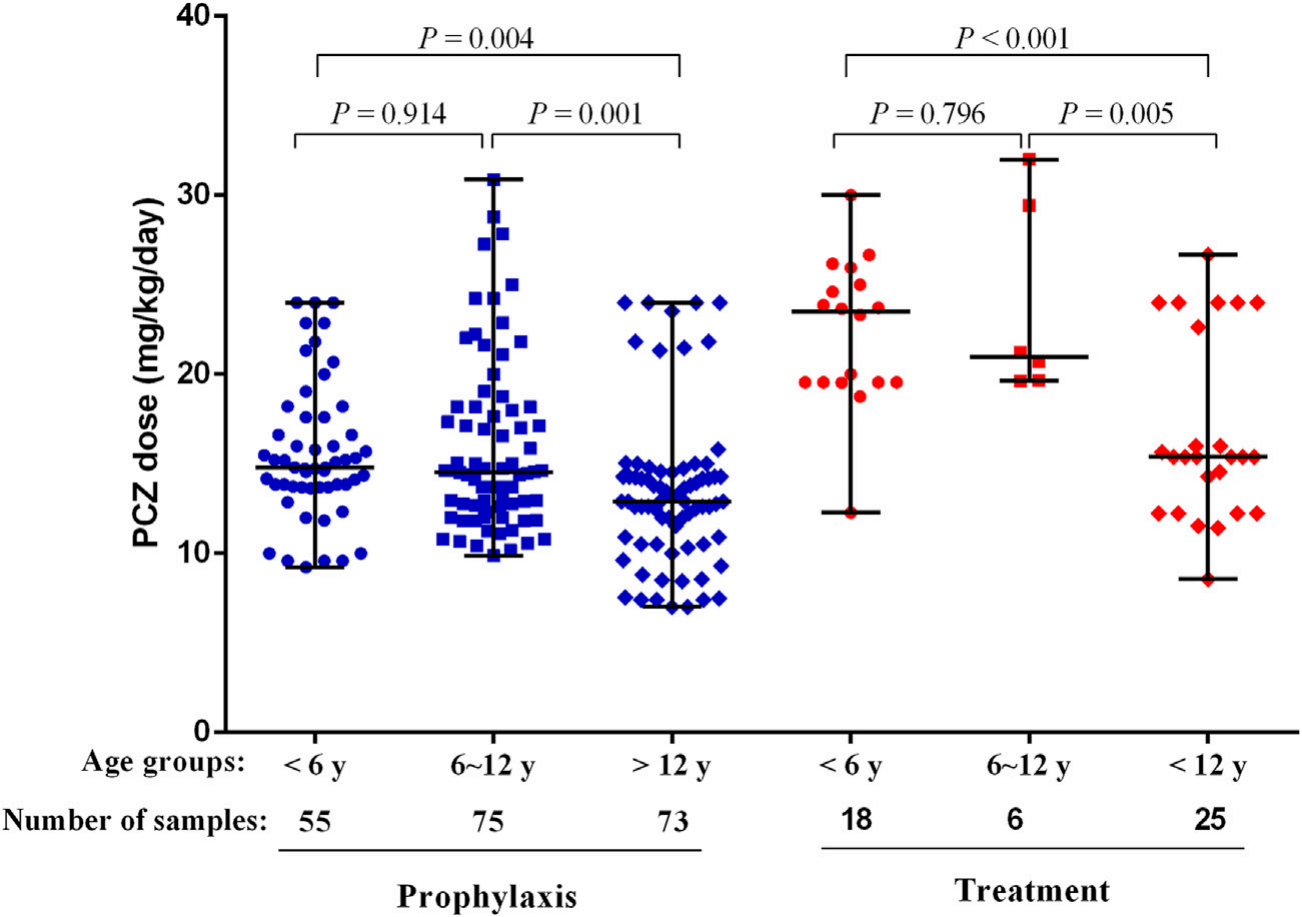
PCZ maintenance dose required to achieve therapeutic Cmin for different age and the indication of treatment. Horizontal bars represent the median dose values for each group. y, years.

The median maintenance PZC doses required to achieve therapeutic *C*_min_ of prophylaxis and treatment were 14.17 mg/kg/day (range, 7.02–30.89 mg/kg/day) and 19.56 mg/kg/day (range, 8.57–32.00 mg/kg/day), respectively (*p* < 0.001). The median maintenance doses required to achieve therapeutic *C*_min_ for different age and the indication of treatment are shown in Figure 4.

Patients received PCZ for prophylaxis of <6, 6–12 and >12 years required median doses of 14.80 mg/kg/day (range, 9.23–24.00 mg/kg/day), 14.52 mg/kg/day (range, 9.88–30.89 mg/kg/day), and 12.90 mg/kg/day (range, 7.02–24.00 mg/kg/day), respectively (*p* = 0.001), to achieve therapeutic *C*_min_. Additional analysis among the three age groups showed that younger patients (<6 and 6–12 years) needed a significantly higher median PCZ dose to maintain the therapeutic trough levels compared to the older children (>12 years) (14.80 versus 12.90 mg/kg/day, 14.52 versus 12.90 mg/kg/day, respectively; *p* < 0.05). In contrast, the median maintenance dose between patients <6 and 6–12 years old did not differ significantly (14.80 versus 14.52 mg/kg/day, *p* = 0.914).

For the children taken PCZ for treatment, the median doses needed by patients <6, 6–12 and >12 years were 23.50 mg/kg/day (range, 12.28–30.00 mg/kg/day), 20.96 mg/kg/day (range, 19.64–32.00 mg/kg/day), and 15.38 mg/kg/day (range, 8.57–26.67 mg/kg/day), respectively (*p* < 0.001), to achieve therapeutic *C*_min_. Additional analysis among the three age groups showed that younger patients (<6 and 6–12 years) needed a significantly higher median PCZ dose to maintain the therapeutic trough levels compared to older children (23.50 versus 15.38 mg/kg/day, 20.96 versus 15.38 mg/kg/day, respectively; *p* < 0.05). In contrast, the median maintenance dose between patients <6 years and 6–12 years did not differ significantly (*p* = 0.796).

There was no significant difference in the dosages of PCZ required to achieve therapeutic *C*_min_ and sub-therapeutic for all children, regardless of the indication of treatment (*p* > 0.05 for all 6 groups). (Table 2).

**TABLE 2:** PCZ dosage and patient information.

	Total	Age		
		<6 years	6–12 years	>12 years
Prophylaxis				
Sample	273	77 (28.2%)	94 (34.4%)	102 (37.4%)
Dose (mg/kg/day)				
≥0.7 μg mL^−1^	14.17 (7.02–30.89)	14.80 (9.23–24.00)	14.52 (9.88–30.89)	12.90 (7.02–24.00)
<0.7 μg mL^−1^	14.02 (5.16–40.00)	17.37 (5.16–37.05)	14.63 (12.00–40.00)	13.19 (6.67–26.67)
Treatment				
Sample	102	31 (30.4%)	32 (31.4%)	42 (41.2%)
Dose (mg/kg/day)				
≥1.0 μg mL^−1^	19.56 (8.57–32.00)	23.50 (12.28–30.00)	20.96 (19.64–32.00)	15.38 (8.57–26.67)
<1.0 μg mL^−1^	23.04 (9.84–32.00)	23.38 (10.29–26.67)	27.45 (16.55–32.00)	21.82 (9.84–28.24)

Data is presented as n (%), or as median (range). y, years.

### PCZ Concentration and Clinical Outcomes

Clinical response was analyzed in patients with administration of PCZ more than 14 days. Among the 78 patients receiving PCZ for prophylaxis of IFIs, 75 patients were identified in the analysis of efficacy of PCZ prophylaxis. 9 of the 75 (12.0%) patients with a median PCZ *C*_min_ of 0.43 μg mL^−1^ (range, 0.13–0.70 μg mL^−1^) developed proven or probable breakthrough IFIs during the study period. These trough levels were significantly lower than the other 66 patients without IFIs (median 1.20 μg mL^−1^, range, 0.12–2.10 μg mL^−1^, *p* < 0.001) (Table 3).

**TABLE 3:** Relationship between outcomes and PCZ Cmin.

Outcome	Prophylaxis			Treatment		
	Sample	Cmin (μg·mL^−1^)	*p*-Value	Sample	Cmin (μg·mL^−1^)	*p*-Value
Success	66 (88.0)	1.20 (0.12–2.10)	<0.001	19 (79.2)	1.06 (0.55–3.08)	0.024
Failure	9 (12.0)	0.43 (0.13–0.70)		5 (20.8)	0.53 (0.30–0.66)	

Data is presented as n (%), or as median (range).

Twenty-four patients were identified in the analysis of efficacy of PCZ treatment. The median PCZ concentration of 1.06 μg mL^−1^ (range, 0.55–3.08 μg mL^−1^) in 19 patients (79.2%) with a positive clinical response was significantly higher than that in the remaining 5 patients (20.8%) with disease progression (median 0.53 μg mL^−1^, range, 0.30–0.66 μg mL^−1^, *p* < 0.024) (Table 3).

**FIGURE 5:**
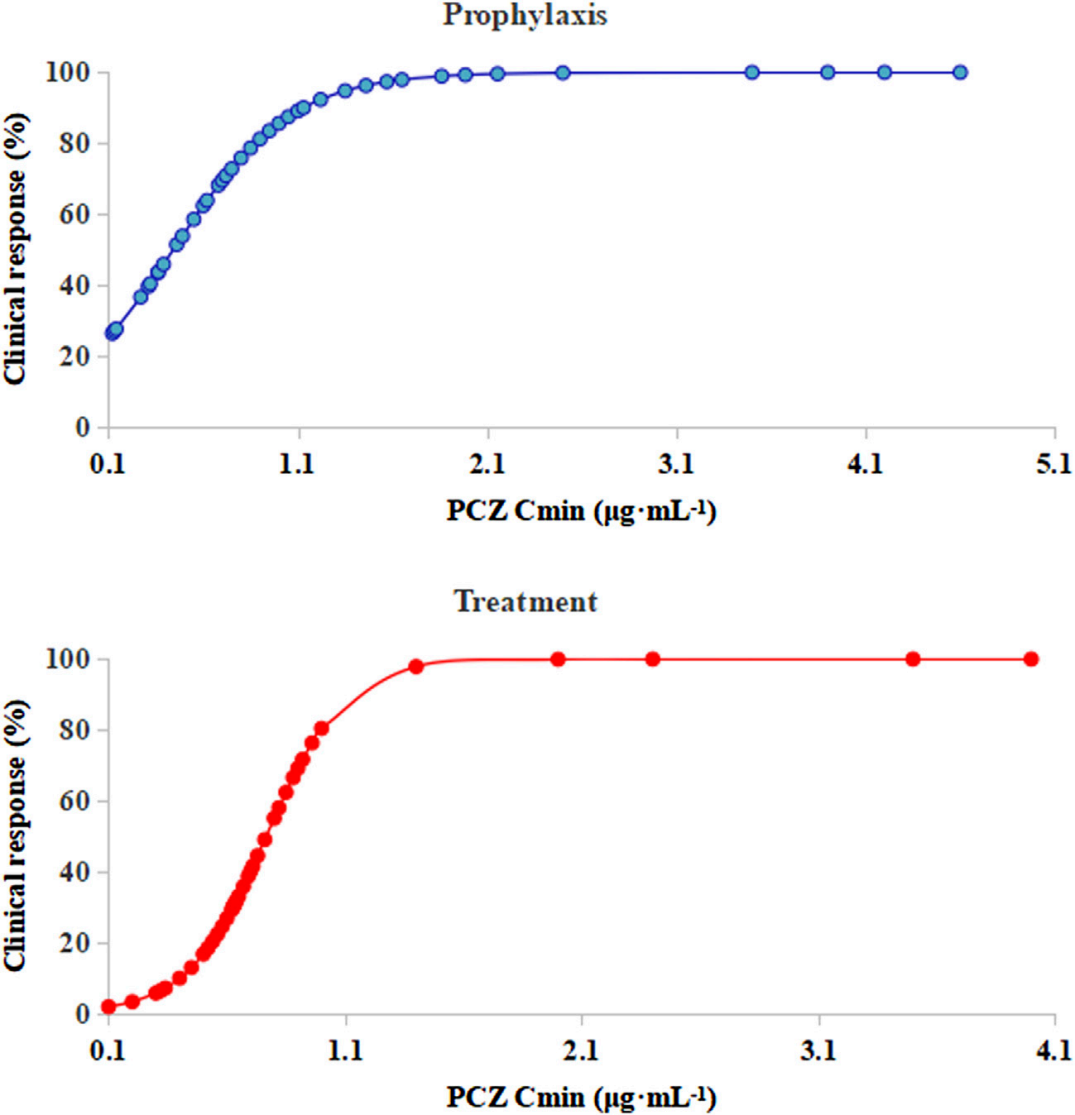
Logistic regression model showing PCZ *C*_min_ predicting the probability of successful clinical response of prophylaxis and treatment. The solid lines represent the regression fit; *C*_min_, posaconazole average steady-state plasma concentrations.

Logistic regression analysis showed that PCZ steady-state average plasma concentrations correlated well with clinical responsiveness. The predicted probability of successful clinical response of prophylaxis and treatment with increasing PCZ concentration from the logistic regression analysis is displayed in Figure 5. Out of 105 patients, 3 (4.76%) died during the observation period, due to relapse of the underlying disease. None of the patients included in the present analysis died from IFIs during the observation period.

### Safety and Tolerability Analysis

Twenty-four experienced hepatotoxicity (24/105, 22.8%). The treatment-related hepatotoxicity emerged by day 12 (range, 3–30) after starting PCZ therapy. Univariate analysis did not reveal any statistically significant association between PCZ trough level and the occurrence of hepatotoxicity (0.95 ± 0.67 μg mL^−1^ versus 1.13 ± 0.58 μg mL^−1^, *p* = 0.369). Three patients discontinued PCZ treatment due to adverse events. These adverse events were considered likely to be related to PCZ treatment for two patients and possibly related (hepatotoxicity and hypokalemia) for one of them. These side effects were always controlled by discontinuing PCZ without any consequences.

### Factors Influencing PCZ Concentration

**FIGURE 6:**
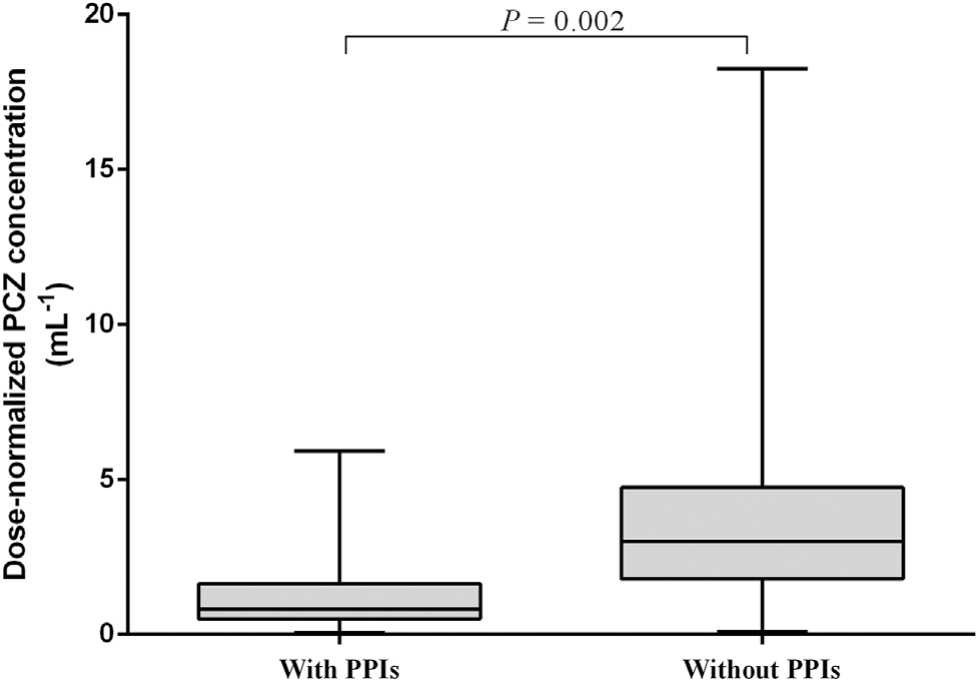
Effect of proton pump inhibitors (PPIs) on dose-normalized PCZ *C*_min_. The graph shows boxplots with whiskers (min to max) of dose-normalized *C*_min_.

Multiple linear regression analysis identified a number of drug interactions and clinical factors associated with a significant change in dose-normalized plasma PCZ concentration (Table 4). Concomitant administration of PPIs had a 64.4% lower blood plasma concentration (median trough concentration, 1.26 versus 3.54 μg mL^−1^, *p* = 0.002) (Figure 6). In addition, patients’ age (*p* < 0.001), CRP (*p* = 0.042), and ALT (*p* = 0.022) were also associated with significantly reduced PCZ concentrations.

**TABLE 4:** Univariate and multilevel linear regression to examine factors influencing dose normalized plasma PCZ concentration.

	Univariate Analysis		Multilevel Linear Regression	
Variable	Coefficient (95% CI)	*p*-Value	Coefficient (95% CI)	*p*-Value
Age	−0.217 (−0.255, −0.178)	<0.001	−0.110 (−0.151, −0.069)	<0.001
Male	−1.380 (−1.938, −0.821)	<0.001		
BMI	−0.293 (−0.368, −0.217)	<0.001		
HSCT	1.258 (0.657, 1.859)	<0.001		
Nausea/emesis	−1.841 (−2.420, −1.261)	<0.001		
Diarrhoea	−1.972 (-3.541, −0.404)	0.014		
Mucositis	−0.716 (−1.974, 0.543)	0.264		
CRP	−0.008 (−0.014, −0.002)	0.010	−0.005 (−0.011, 0.000)	0.042
Pct	−0.027 (−0.067, 0.012)	0.172		
GFR	−0.036 (−0.047, −0.025)	<0.001		
Albumin	0.044 (−0.007, 0.096)	0.093		
ALT	−0.008 (−0.013, −0.003)	0.003	−0.004 (−0.008, −0.001)	0.022
AST	−0.001 (−0.008, 0.008)	0.997		
g-GT	−0.009 (−0.012, 0.005)	<0.001		
ALP	0.001 (−0.003, 0.005)	0.613		
TBIL	−0.037 (−0.054, −0.020)	<0.001		
Concomitant medications				
PPIs	−2.160 (−2.689, −1.631)	<0.001	−0.857 (−1.387, −0.327)	0.002
Metoclopramide	0.633 (0.112, 0.845)	0.155		
Phenytoin	−2.473 (−7.406, 2.460)	0.325		
rifampicin	−1.299 (−3.177, 0.578)	0.174		

HSCT, Allogeneic hematopoietic stem cell transplantation; CRP, C-reactive protein; Pct, Procalcitonin Creatinine clearance; GFR, Glomerular filtration rate; PPIs, Proton pump inhibitors; ALT, Alanine transaminase; AST, Aspartate transaminase; g-GT, g-Glutamyltranspeptidase; ALP, Alkaline phosphatase; TBIL, Total bilirubin. Bold formatting signifies statistically significant covariates.

## Discussion

We reported on the relationship between the PCZ therapeutic drug monitoring and dose in immunocompromised children. It is difficult to attain target concentrations in children, especially in those <13 years old and there is a significant variability in the dose required as a result of unpredictable bioavailability. A large interindividual variability in PCZ *C*_min_ was observed which cannot be explained by the dose-concentration relationship. This study demonstrated that dose-normalized serum concentrations of PCZ were affected by age, CRP, ALT, and coadministration of PPIs (Table 4).

The pharmacokinetics of PCZ in children has not been fully studied to date, and limited information is available on optimal dosing regimen for oral suspension for pediatric patients. Mathew et al. examined thirty-two immunocompromised children (aged 3–18 years) receiving PCZ for both prophylaxis and treatment (Mathew et al., 2017). Sixteen of thirty-two patients (50%) achieved target PCZ (0.7 μg mL^−1^) with a median initial dose of 22.8 mg/kg/day (range, 9.2–44 mg/kg/day). The remaining sixteen patients did not achieve PCZ target with a median initial dose of 15.8 mg/kg/day (range, 9.3–50 mg/kg/day). Vicenzi et al. assessed the correlation between PCZ daily dose and trough plasma level in hematology-oncology pediatric patients (Vicenzi et al., 2018). For the prophylaxis group, a median dose of 12 mg/kg/day (range, 4–35 mg/kg/day) of PCZ oral solution was associated with a PCZ *C*_min_ > 0.7 μg mL^−1^ in 63% of PCZ assessments at day ≥7 after starting PCZ. In the group of patients which used PCZ as salvage therapy for IFI, a median dosage of 15 mg/kg/day (range, 5–30 mg/kg/day) was associated with the achievement of a threshold above 1 μg mL^−1^ in 77% of measurements performed in the first week of treatment.

In our study, the median PCZ dose for prophylaxis was 14.80 mg/kg/day for patients <6 years, 14.52 mg/kg/day for patients 6–12 years and 12.9 mg/kg/day for patients >12 years, respectively. The median dose for treatment was 23.50 mg/kg/day for patients <6 years, 20.96 mg/kg/day for patients 6–12 years and 15.38 mg/kg/day for patients >12 years, respectively (Table 2). Higher PCZ doses were required to achieve the target *C*_min_ among younger children. However, the median maintenance dose did not differ significantly between patients aged <6 and 6–12 years old in this study, although a higher trend is shown in patients <6 years old (Figure 4). One possible explanation for the higher doses in patients <13 years is that patients in this group were dosed based on current available data versus patients 13 years and older who were dosed based on package insert recommendations (Bernardo et al., 2020).

Few trials have described PCZ *C*_min_ and efficacy relationgship in pediatric patients and no guidelines are available regarding the target PCZ plasma concentration for pediatric patients currently. Dolton et al. reported that among 72 patients taking PCZ for prophylaxis against IFIs, and 12 patients (17%) developed a breakthrough fungal infection (Dolton et al., 2012); the median PCZ concentrations were significantly lower than those who did not (0.289 versus 0.485 μg mL^−1^; *p* < 0.01). In the present study, a significant exposure-response relationship was observed. In the prophylaxis group, all breakthrough fungal infections were observed at the median PCZ concentrations below 0.50 μg mL^−1^, suggesting that this cutoff value may be a useful concentration target for PCZ prophylaxis against IFIs in children, which is considerably lower than described in our previous study in adult patients with hematologic disorders (0.76 μg mL^−1^) (9). In that analysis, a cut-off *C*_min_ was identified for patients as the concentration where successful clinical response increased >80% probability (Wang et al., 2014).

Similar to a retrospective study of PCZ prophylaxis of IFIs in pediatric patients with hematology oncology [the success rate was (80/84) 95.2%], and a randomized clinical trial for prophylaxis against IFIs [the success rate was (50/70) 71.4%], the current study found that 88.0% of patients (66/75) successfully responded to PCZ prophylaxis (Vicenzi et al., 2018; Sienkiewicz-Oleszkiewicz et al., 2019). Among patients receiving PCZ for the treatment of IFI, PCZ concentrations among patients who failed therapy (0.53 μg mL^−1^) were lower than those who treated successfully (1.06 μg mL^−1^) (*p* = 0.024). In our previous study, PCZ *C*_min_ values of 1.0 μg mL^−1^ was associated with a >80% probability of successful treatment response (Jia et al., 2020). Similar to a multicenter study of PCZ treatment of IFIs in juvenile (<18 years) with IFIs [the success rate was (5/8) 62.5%] (Krishna et al., 2007), the current study found that 79.2% of patients (19/24) successfully responded to PCZ treatment. Taken together, the relationship between PCZ *C*_min_ and efficacy may follow a different profile among pediatric patients. Therefore, a lower limit of the target *C*_min_ should be considered for Asian children compared with adults. This retrospective study is the first to assess the relationship between PCZ *C*_min_ and efficacy in children. Future studies of larger patient cohorts are needed to accurately define concentration targets for PCZ in the prophylaxis and treatment of IFIs.

In our study, PCZ was well tolerated in children, and the rate of hepatotoxicity possibly related to PCZ was 22.8% and all reverted with the discontinuation of the drug, comparable to previously reported rates (3.0–16.7%) (Döring et al., 2017; Vicenzi and Cesaro, 2018). Similar to previous reports, we found no consistent correlation between PCZ trough concentrations and hepatotoxicity (Krishna et al., 2007). Because PCZ concentrations are relatively constant at steady state, the average of all plasma concentrations for each patient was calculated to provide a single steady-state plasma PCZ concentration, which was used to analyze the association of *C*_min_ with efficacy and safety.

Concomitant PPI therapy has been shown to be significantly associated with decreased concentrations in adult patients (Jia et al., 2020; Krishna et al., 2009). In a population-pharmacokinetic study in children, Boonsathorn et al. found a 42% reduction in relative bioavailability in patients taking PPIs (Boonsathorn et al., 2019). Co-administration of PPIs was associated with a 64.4% reduction in PCZ plasma concentration in our study (Figure 6). It is unlikely that this interaction is cytochrome P450 mediated because PCZ undergoes limited metabolism primarily by UDP-glucuronosyltransferase UGT1A4 (Lipp, 2010). The later study also confirmed that the pH-dependent solubility of PCZ through monitoring of intraluminal PCZ concentrations (Walravens et al., 2011). We did not observe phase II glucuronide enzyme inducers such as phenytoin or rifampicin to be significantly associated with dose-normalized PCZ concentration (Table 4), probably due to the low samples taken concurrently with these drugs (0.5 and 2%, respectively). The univariate and multiple linear regression analysis both showed that lower PCZ concentrations were observed with increasing CRP concentrations. However, the effect is less distinct compared to adult patients (Jia et al., 2020). Therefore, therapeutic drug monitoring of PCZ should be warranted in pediatric patients with elevated CRP level.

There are limitations in our study. This was a single-institution retrospective study, and polymorphisms that could influence plasma PCZ concentration were not checked. In addition, TDM of PCZ was not routinely performed during the study period (especially low numbers of cases in children with IFI). Further pediatric studies are needed to confirm the results of the present study, and especially to standardize TDM and dose adjustment of PCZ to improve attainment of target concentrations.

## Conclusion

This study confirmed that age, CRP, ALT and co-administration of PPIs were independent factors affecting PCZ exposure in children. In prophylaxis group, therapeutic concentrations of PCZ can be obtained in the majority of children with a dose of 14.17 mg/kg/day. In the treatment groups, the dose was 19.56 mg/kg/day. Younger patients might need a higher dosage of PCZ to achieve target concentrations compared to older children.

## Data Availability

The raw data supporting the conclusions of this article will be made available by the authors, without undue reservation.
